# Glacial melting: an overlooked threat to Antarctic krill

**DOI:** 10.1038/srep27234

**Published:** 2016-06-02

**Authors:** Verónica Fuentes, Gastón Alurralde, Bettina Meyer, Gastón E. Aguirre, Antonio Canepa, Anne-Cathrin Wölfl, H. Christian Hass, Gabriela N. Williams, Irene R. Schloss

**Affiliations:** 1 Instituto de Ciencias del Mar (CSIC), Barcelona, Spain; 2 Consejo Nacional de Investigaciones Científicas y Técnicas (CONICET), Argentina; 3 Instituto de Diversidad y Ecología Animal (IDEA), CONICET-UNC y Facultad de Ciencias Exactas, Físicas y Naturales, Universidad Nacional de Córdoba, Córdoba, Argentina; 4 Alfred Wegener Institute, Helmholtz Centre for Polar and Marine Research, Bremerhaven, Germany; 5 Institute for Chemistry and Biology of the Marine Environment, Carl von Ossietzky University of Oldenburg, Oldenburg, Germany; 6 School of Marine Sciences, Pontificia Universidad Católica de Valparaíso, Valparaíso, Chile; 7 Alfred Wegener Institute Helmholtz Centre for Polar and Marine Research, Wadden Sea Research Station, List, Germany; 8 Centro para el Estudio de Sistemas Marinos, Puerto Madryn, Chubut, Argentina; 9 Instituto Antártico Argentino, Buenos Aires, Argentina; 10 Institut des sciences de la mer de Rimouski, Rimouski, Quebec, Canada

## Abstract

Strandings of marine animals are relatively common in marine systems. However, the underlying mechanisms are poorly understood. We observed mass strandings of krill in Antarctica that appeared to be linked to the presence of glacial meltwater. Climate-induced glacial meltwater leads to an increased occurrence of suspended particles in the sea, which is known to affect the physiology of aquatic organisms. Here, we study the effect of suspended inorganic particles on krill in relation to krill mortality events observed in Potter Cove, Antarctica, between 2003 and 2012. The experimental results showed that large quantities of lithogenic particles affected krill feeding, absorption capacity and performance after only 24 h of exposure. Negative effects were related to both the threshold concentrations and the size of the suspended particles. Analysis of the stomach contents of stranded krill showed large quantities of large particles ( > 10^6 ^μm^3^), which were most likely mobilized by glacial meltwater. Ongoing climate-induced glacial melting may impact the coastal ecosystems of Antarctica that rely on krill.

In the summer of 2002, a massive stranding event of the tunicate *Salpa thompsoni*, and the euphausiid *Euphausia superba* (hereafter referred to as ‘krill’), which are key components of the Southern Ocean ecosystem, was observed in front of the Argentinean Antarctic Station Carlini (formerly known as Jubany Station) along the shore of Potter Cove (King George Island/25 de Mayo Island, South Shetland Islands, Fig. 1a)^1^. We suspected that high concentrations of suspended particulate material, primarily of glacial origin, may have been associated with this stranding event. Since that event, the beaches of Potter Cove have been surveyed, and repeated stranding events have been recorded (Table 1).

Potter Cove is surrounded by the Fourcade Glacier to the North and the East. A detailed description of the area has been published^2^. The glacier has been receding at an increasing rate (up to 1 km since the 1950s^3^), a trend that has also been observed for other glaciers on King George Island^4^. Glacial retreat causes the massive discharge of sediment-laden meltwater. This discharge was observed in Potter Cove during the summer for more than 20 years^2^. Sediment-laden surface water plumes, called “brown waters”, originate from meltwater creeks as well as from the glacier itself (Fig. 1b). They are a common feature in the area^5^,^6^, and the transport of sediments to the sea is a natural process in coastal areas, which occurs each spring. In Maxwell Bay, sediment mass accumulation rates of up to 0.66 g cm^−2^ yr^−1^ have been documented during the last 100 years^7^. Due to the present warming, dramatic increases in glacial melting have resulted in a rising particle discharge via surface and sub-surface drainage. This particle discharge is recognized as a threat to coastal ecosystems^8^,^9^.

Large quantities of suspended particles affect the growth and survival of benthic filter feeders^10^,^11^ by clogging their filtration systems^8^. In copepods, inorganic particles affect feeding efficiency, carbon turnover and egg production^12^ as well as mortality rates^13^. Deleterious effects such as reduced foraging, growth and changes in physiological condition have also been observed in fish^14^.

The Antarctic krill is a key species in the Southern Ocean trophic web^15^ and to the biological CO_2_ pump in the continental shelf of the Western Antarctic Peninsula (WAP) through the production of faecal pellets and the export of particulate carbon to deeper waters^16^. A strong decline in krill since the mid-1970 s has been associated with changes in sea ice^17^.

Potter Cove is a tributary fjord of Maxwell Bay in the southern coast of King George Island, South Shetland Islands, and is 4.7 km long and 1.6 km wide. A high krill abundance is characteristic of the oceanic waters off the South Shetland Islands and of spawning areas such as the Scotia Sea (average density approximately 25–50 and 50–100 ind m^−2^, respectively^18^). However, in nearshore waters, such as off Livingston Island, krill biomass densities have been shown to be higher, more stable and less variable than in the Scotia Sea^19^. To date, specific studies on krill abundance have not been conducted in bays such as Potter Cove and others along the WAP. However, the presence of krill is suggested by the stomach contents of the fish *Notothenia coriiceps*, the dominant benthic fish in Potter Cove^20^, which feeds primarily on krill^21^, and by the presence of whales feeding on krill inside the cove, as described for other coastal bays in the WAP^22^. Moreover, compared with the shelf and the slope, the coastal WAP showed a higher krill abundance between 1993 and 2013^23^.

In the majority of the stranding events documented at Potter Cove since 2002, krill was the primary species found. Therefore, our aim was to understand the effect of the concentration and size of suspended particles on krill feeding, food absorption efficiency and survival. Here, we provide evidence showing that the quantity and quality of suspended inorganic particles are the cause of the mass krill strandings observed, providing the first insight into a phenomenon that may be crucial to the polar coastal marine food web in a warming world.

## Results

### Environmental conditions during krill stranding events

**Figure 2:**
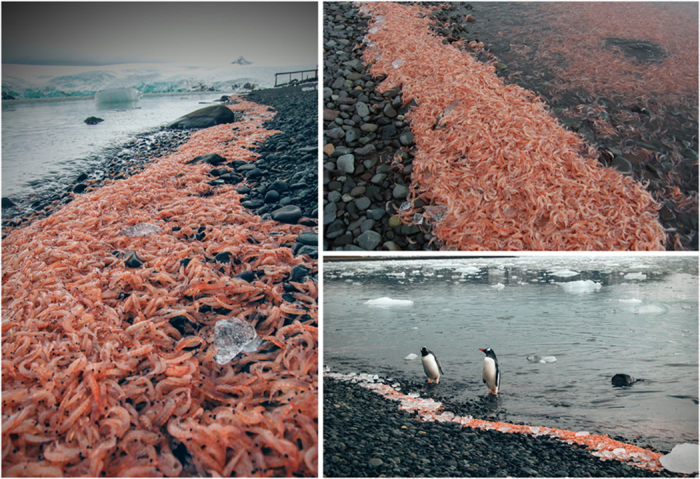
Stranded krill on the beaches of the southern shore of Potter Cove. Credit photos: V. Fuentes.

In the austral summer of 2003, four krill stranding events were detected on Potter Cove’s southern shore, but only 3 were quantified (the fourth event was documented after most of the krill had drifted out to sea or had been ingested by birds). The average density of dead krill on the beach in these events was 1,457 ± 740 ind m^−2^ of beach surface, of which 20% were adult krill, 2% were juveniles and 78% were larval krill; the total number of dead krill on the beach ranged between 130,000 and 413,000 individuals. The largest stranding event was observed in March 2007 (1,042 ± 277 ind m^−2^, 93% juveniles, 7% adults) along a 400 m coastline (Fig. 2) with a median total of approximately 423 000 individuals (range: 307,000 to 521,000). The environmental conditions during each of the recorded events are summarized in Table 1. Prior to the stranding events, the total amount of suspended particulate matter was similar for all documented events (*p* = 0.976) and was positively correlated with both the number of positive degree-days (PDDs) (t_(23)_ = 2.604, *p *< 0.050) and the maximum tide amplitude (t_(23)_ = 2.623, *p *< 0.050). Generalized linear models (GLMs) indicated that the probability of mass (≥100 ind m^−2^, category 2, Table 1) strandings increased directly with air temperature and maximum tidal amplitude. When a 24 h time lag was taken into account, total suspended particulate matter (TSPM) and wind speed showed a unimodal response such that high values (of both variables) were positively correlated with the probability of mass strandings. Not only was the maximum wind speed (>25 knots on average) important, but the dominant wind direction 24 h prior to the observed krill stranding events was also important (*p *< 0.050). Wind direction associated with category 2 events typically originated from the N and NW quadrants. Furthermore, when air temperature was >0 °C, a positive correlation with the strandings was evident.

### Impact of the quantity of sediment on krill feeding activity

**Figure 3:**
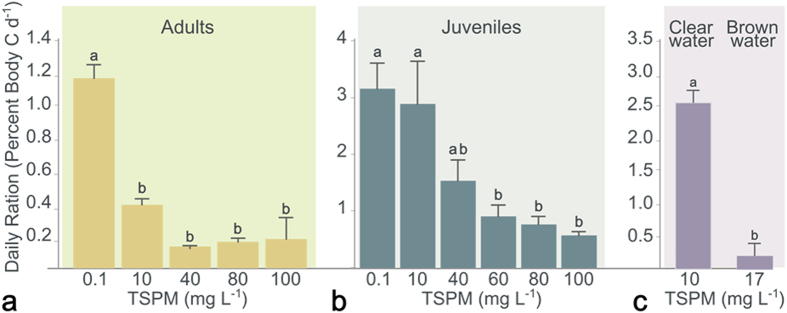
Daily rations of Antarctic krill fed with varying quantities of particles. Two sets of experiments were run. The first had bottom sediments added to the cultured phytoplankton; (**a**) Adults, (**b**) Juveniles. The second (**c**) used natural waters, i.e., clear water with no evident particles present in the water and brown water with a heavy load of particles.

Krill showed a linear increase in feeding rate in response to increasing concentrations of natural phytoplankton cultures (with no added suspended particulate matter). The maximum feeding rate (14% body C d^−1^) was obtained at phytoplankton concentrations of 400 μg C L^−1^. In Potter Cove, the most frequent phytoplankton concentration ranged between 30 and 60 μg C L^−1^. Krill feeding on phytoplankton within this concentration range (which corresponds to our control treatment) showed an average daily carbon ration of 1.19 ± 0.10% body C d^−1^. In the feeding experiments with different quantities of added bottom sediment from Potter Cove (10, 40, 60, 80 and 100 mg TSPM L^−1^), even the lowest sediment concentration significantly decreased (*p *< 0.050) the daily carbon ration of adult krill to 0.42 ± 0.03% body C d^−1^ (Fig. 3a). There were no significant differences in the daily carbon ration among the different concentrations of added sediment (*p *< 0.050). In juveniles, the daily carbon ration was similar to the control for added sediment concentrations up to 40 mg TSPM L^−1^ and only decreased significantly at concentrations greater than 60 mg TSPM L^−1^ (*p *< 0.050, Fig. 3b). Shortly after the sediment was added (particle size <50 μm; see methods) to the phytoplankton, the digestive tract of the krill became a brown colour, indicating that the sediment had been ingested by the krill; this effect was similar to *in situ* observations of krill feeding in sediment-laden waters of Potter Cove (see supplementary material). When the feeding experiments were performed with *in situ* ‘brown water’ from Potter Cove (0.9 μg Chl-*a* L^−1^ and 17 mg TSPM L^−1^), the daily carbon ration dropped significantly, by more than 80% (*p *< 0.001) compared with experiments using clear water (Fig. 3c), suggesting that the quality and the size range of TSPM had an impact on krill feeding.

### Absorption efficiency of krill in the presence of sediments

**Figure 4:**
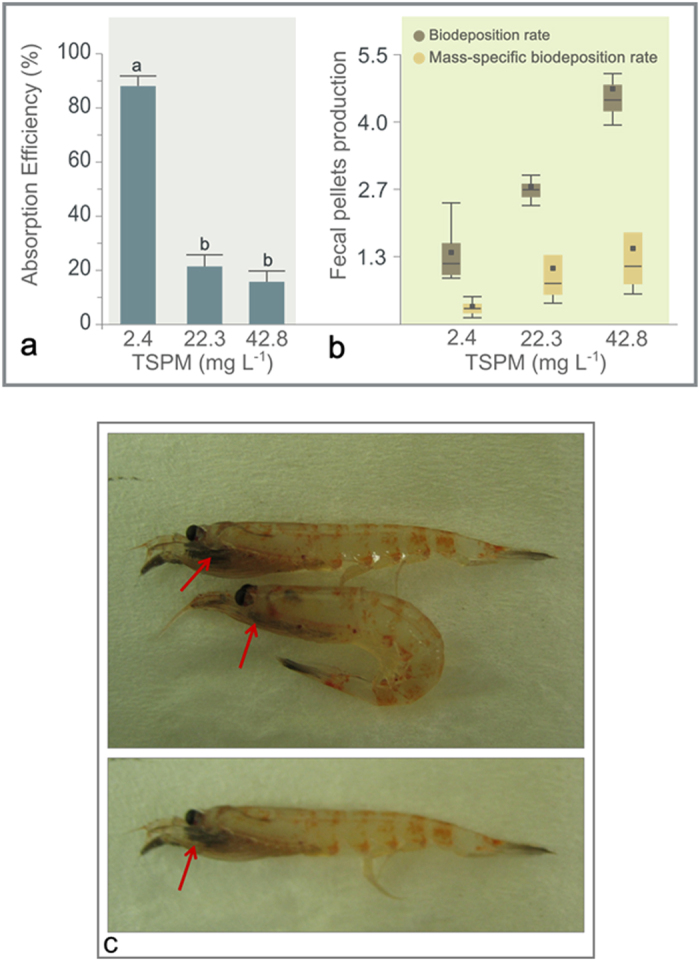
(**a**) Absorption efficiencies and (**b**) biodeposition rates (in mg fecal pellets ind^−1^ and mass-specific biodeposition rates (in mg fecal pellets g ind^−1^) of krill fed with natural seston to which two different amounts of sediments were added. TSPM: Total suspended particulate matter. (**c**) Pictures of krill after the feeding experiments with high concentrations of suspended particulate material. Red arrows point to the particles in the feeding apparatus of the animals. Credit photos: V. Fuentes.

When juvenile krill fed only on natural phytoplankton cultures, in the absence of sediments, the absorption efficiency reached 88.29 ± 3.72%, whereas when fine (<50 μm) bottom-sediment was added (20 and 40 mg TSPM L^−1^), the absorption efficiency decreased to 21.44 ± 15.27 and 15.75 ± 3.62%, respectively (Fig. 4a). Once the sediment was added to the phytoplankton, faecal pellet production increased with increasing quantities of suspended particulate material (Fig. 4b). The rates of biodeposition (mg faecal pellets ind^−1^) and mass-specific biodeposition (mg faecal pellets g ind^−1^) were higher at higher particle concentrations (*p *< 0.001). Again, it was observed that the entire digestive system of krill exposed to increased quantities of particles was coloured brown. Moreover, at concentrations of 40 mg L^−1^ TSPM in both sets of experiments, 80% of animals stopped swimming and remained at the bottom of the tanks. Their feeding baskets and guts were brown-coloured (Fig. 4c) with sediments particles adhering to the surface, very probably affecting their capacity for feeding.

### Analyses of krill from 2008 and 2009 stranding events

**Figure 5:**
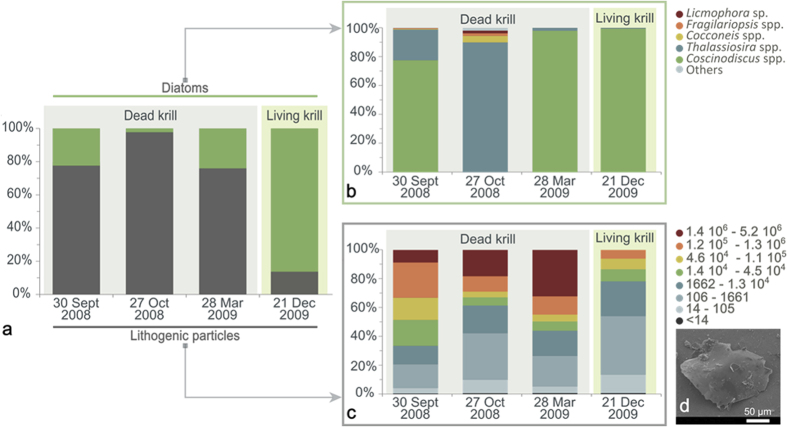
(**a**) Proportion of inorganic particles and diatoms in the gut contents of stranded krill from 2008 to 2009. (**b**) Size classes of the lithogenic particles found in living krill and during the analysed strandings. (**c**) Relative contributions of different diatom species. Note the dominance of the planktonic genera *Coscinodiscus* sp. and *Thalassiosira* sp. (**d**) The relatively sharp angles of the particles as seen under the SEM, indicative of local and relatively recent origin. Credit photos: G. Aguirre.

The entire digestive system of the stranded krill collected during 2008 and 2009 was filled with brown particles, and the digestive gland was a pale yellow-grey colour. Body and stomach wet weights showed no significant differences between the different mortality events (*p* = 0.083 and *p* = 0.490 for body and stomach, respectively, Table 2) and were similar to the body and stomach weights of living krill collected from net tows in clear waters of Potter Cove (Fig. 5a). The digestive glands in living krill had a greenish colour, suggesting ingestion of phytoplankton. Centric pelagic and some benthic, pennate diatoms were the major items in the stomach contents of these animals (Fig. 5b). Diatoms were also present in the digestive tract of dead krill; total diatom volume did not vary among mortality events (*p *< 0.050) (Table 2) but was significantly lower than in the stomachs of living krill (Mann-Whitney U = 16; *p *< 0.001). However, highly significant differences were found in the total amount of lithogenic particles between dead and living krill (*p *< 0.001) in both years. Dead krill had more than two times the volume of lithogenic particles in their digestive tract than living krill. Large irregular lithogenic particles (>1.1 10^5 ^μm^3^ volume, equivalent to >75 μm diameter) represented a large fraction of the contents of the digestive tract in dead krill for all three mortality events in the 2008 and 2009 seasons, and no significant difference in the volume of lithogenic particles in dead krill was found between these events (*p* = 0.840). Very large particles, in the 1.4 10^6^–5.2 10^6 ^μm^3^ range (175–275 μm length), were only found in the stomachs of krill from the mass mortality events (Fig. 5c).

## Discussion

Mass mortality events of marine organisms are receiving increasing attention. A comprehensive revision has been recently published^24^. The presence of particulate matter has been invoked as the cause for salp and copepod deaths in coastal environments^1^,^13^.

In light of our findings, we have strong evidence that the quantity and quality of lithogenic material in the water column can be a significant threat for krill in the shallow coastal regions of the Antarctic Peninsula. We postulate that the ingestion of large lithogenic particles (>1.1 10^5 ^μm^3^), which are most likely of glacial origin, is the primary cause of the krill mass mortalities studied. This study is the first documentation of the impact of large quantities and large sizes of lithogenic particles on krill.

Krill is a very effective filter feeder^25^ adapted to food sources of varying size and density^15^. They have often been observed feeding near sea floor sediments in coastal areas^26^ and have been recorded feeding in meltwater plumes (see supplementary material). Stomach content analyses suggest that krill are adapted to sporadically cope with a certain level of sediment, as evidenced by the gut content analysis of living krill, both here and in previous studies (e.g., 26). The results from our feeding experiments, in which fine bottom sediments from Potter Cove were added to natural plankton, demonstrate that this type of material, even in low concentrations (10 mg L^−1^ TSPM), has a strong impact on the performance of adult krill. Juveniles showed a similar response at particle concentrations >60 mg L^−1^. The difference between these age groups may be due to the mean mesh interval of their filtration apparatus. For juvenile krill, this ranges between 19 and 36 μm^27^, which is smaller than the maximum size of the bottom sediment particles used in these experiments. It is probable that most particles did not enter their digestive tract. However, at high concentrations, their filtering basket gets clogged (see Fig. 4c). By contrast, the mean mesh interval of adult krill ranges from 68 to 83 μm, such that 50 μm particles were not filtered out and immediately entered their digestive tract, severely affecting their body carbon ration and hence their overall performance, as evidenced by the cessation of movement and their position at the bottom of the tank. However, the feeding experiments with “brown water” from Potter Cove that originated from glacial melting showed that even a low particle concentration (17 mg L^−1^ TSPM) had a serious negative impact on the daily carbon ration and overall condition of juvenile krill. This impact was similar to the experiment in which 100 mg L^−1^ TSPM of fine bottom sediment was added. The mean grain size of the sediment transported via glacier meltwater into the cove ranges from 6 to 781 μm^6^. The size of the bottom sediments used for the experiment was <50 μm. Therefore, according to our experimental results, in addition to the quantity of TSPM, the quality of TSPM is also probably having an impact on the daily carbon ration and performance of krill. Moreover, when particles were added to the food, an immediate increase in faecal pellet production was noted, which diminished the gut residence time.

Due to the morphology and the function of specific organs, the krill’s ability to process lithogenic particles is limited. Before large cells can be ingested, the mandibular *pars molaris* of krill split diatom chains and cut or fracture hard particles^27^. Ingested solid food particles are further macerated and crushed by the gastric mill, which is located inside the stomach, and mixed with digestive enzymes^28^,^29^. The processed material is then pressed through a fine filter system that allows fine food particles (<0.2 μm) to enter the midgut, where they are further digested. It is probable that the gastric mill of krill is not able to crush and grind lithogenic particles. Although coarse food residues, together with small lithogenic particles, can be transported directly to the hindgut^25^, large lithogenic particles may not enter the hindgut due to their size. This may represent an interruption of the passage of ingested particles, suggesting that the ingestion of large lithogenic particles (>1.1 10^5 ^μm^3^) could also mechanically disrupt the krill intestine. Lithogenic particles were found in the stomach contents of both dead and live krill but represented only a small proportion of the stomach contents of living krill. In addition, no particles >1.3 10^6 ^μm^3^ (175 μm length) were found in live krill. The large size and shape of the lithogenic particles found in the stranded krill guts suggest that this is relatively recent material of glacial origin, with sharp angles compared with particles from the bottom. The diatom species composition in dead krill guts showed a dominance of pelagic diatoms (*Coscinodiscus* spp. and *Thalassiosira* spp., Fig. 5b), indicating that the lithogenic particles in the water column are of glacial origin rather than resuspended from the bottom of the Cove.

Krill can normally avoid unfavourable environments. At Potter Cove, strong and long-lasting N and NW winds are able to build up significant waves that strike the beach at high frequency, hindering the rapid deposition of glacial particles and increasing resuspension in the wave zone. These winds force the meltwater plumes to the southern shore, further increasing the local particulate material concentration in the water^5^. Under such conditions, krill may become trapped in the surf zone close to the beach, ingesting whatever material is present there. Therefore, according to our findings, the consequences for krill are twofold: first, krill cannot cope with a large concentration of lithogenic particles and their filter system becomes clogged, and second, the large quantity of large lithogenic particles interrupts the passage of ingested particles, the accumulation of food in the stomach and hence nutrient absorption. The krill become weak and are easily transported by the waves onto the beach or die in the water.

**Figure 6:**
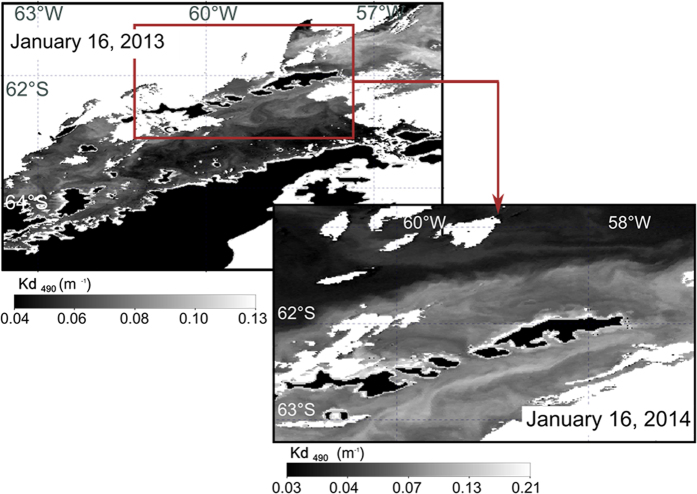
Diffuse attenuation coefficient, Kd, which is strongly correlated with inorganic suspended matter in the water column^36^, as obtained from NASA Goddard Space Flight Center, Ocean Ecology Laboratory, Ocean Biology Processing Group. Moderate-resolution Imaging Spectroradiometer (MODIS) Aqua Ocean Color Data; 2014 Reprocessing. NASA OB.DAAC, Greenbelt, MD, USA. doi: 10.5067/AQUA/MODIS_OC.2014.0. (Accessed on 12/10/2015). MODIS satellite images of the northern WAP and King George Island. Land is shown in black and clouds in white, and Kd is grey scaled. (**a**) January 16, 2013, showing relatively high values of Kd along the coasts of King George Island and lower values along the WAP coast. (**b**) January 16, 2014, showing a detail of the King George Island area, with relatively high values of Kd. In this image, the WAP is covered by clouds and therefore not visible to the satellite and has therefore been omitted. Different dates are presented to highlight the persistence of the phenomenon. Note the difference in Kd scales between images.

Earlier studies have documented the presence of particle-laden “brown water” in Potter Cove^5^. During the storms that occur throughout the active glacial melting periods, the concentration of particulate matter can be >100 mg L^−1^ 10. Over the last six decades, 87% of 244 studied glacier fronts have retreated along the WAP^30^, increasing the discharge of glacial meltwater and particles into the coastal marine ecosystem^2^. Approximately 90% of King George Island’s surface is covered by glaciers that are undergoing the same process, increasing the runoff of meltwater into the bays and fjords of the island^31^,^3^,^4^. The size and angular shape of the particles recovered from stranded krill guts in this study (Fig. 5d) indicate a relatively short transport time of the particles and therefore a local origin, most likely directly from the Fourcade Glacier and its associated meltwater streams. These glacially derived particles exceed the average grain size of particles deposited on the seafloor in Potter Cove^6^, suggesting special discharge events. However, the quantity of material discharged by meltwater forms conspicuous sediment plumes that become part of the coastal waters and can be traced for many tens of kilometres^32^. High diffuse attenuation coefficients (Kd) in coastal polar areas have been correlated with the presence of suspended inorganic particles^33^. The extension of these highly turbid waters can be clearly seen in summer along the northern WAP, particularly near the coasts such as those around King George Island (Fig. 6). These plumes show the potential extent of the area over which krill survival could be threatened. Indeed, the area around Antarctica that is estimated to be affected by changes in the extension of tidewater glaciers alone and their impacts on the biota is 2.97 × 10^6^ km^2^ 9.

An increase in meltwater runoff will additionally reduce surface water salinity and could trigger osmotic stress, as observed for phytoplankton^34^ and amphipods in coastal Arctic waters^35^. However, krill are osmoconformers, and salinities in the range of 25 to 45 psu have little effect on their metabolism^36^. Surface water warming could be an additional source of stress for krill. However, early experiments with temperatures varying from −1 to 10 °C have shown that krill are able to tolerate some exposure to high temperatures by lowering their metabolic rate^37^. The mortality observed in this study should therefore not be related to these stressors but to the amount and the size of glacially derived lithogenic particles. A similar impact was observed on copepods and amphipods in Arctic fjords^38^. Furthermore, krill strandings were correlated with strong N or NW winds in combination with maximum tidal amplitudes. These conditions would lead krill to ingest large quantities and large sizes of lithogenic particles, debilitating the krill. The particle shape further suggests that the lithogenic material is recently derived from the Fourcade Glacier and its associated meltwater streams.

The poor record of krill strandings and the relatively low number of stranded animals would indicate that these are rare events. However, the probability of detecting a stranding event depends on specific physical conditions that push the dead organisms onto the beaches. The number of strandings we observed probably reflects only a small fraction of all the sediment-related krill mortality events, as most coastal areas in Antarctica are not monitored. Most of these dead krill will eventually settle on the sea floor to become part of the benthic food web, thereby remaining undocumented. The importance of dead krill as a source of organic matter for benthic organisms has been previously documented^39^. In addition, the delay between a stranding and its observation is also crucial for the quantification of dead animals. Not only will tides and waves wash the dead organisms out back to the sea, but also animals such as birds will also profit from this easily available food source.

Environmental conditions around Antarctica are changing; in particular, positive air temperature, related to glacial melting, has increased and westerly winds, which are related to the krill stranding events in Potter Cove, have become more frequent in recent decades^40^. The Western Antarctic Peninsula (WAP) is one of the most rapidly warming areas on Earth^41^, and krill is the most abundant species in the coastal areas of this region^23^; predators such as fish, seabirds, penguins, seals and whales rely on this single resource. The presence of numerous krill-feeding whales in fjords, such as Potter Cove and others along the WAP, as well as the presence of the dominant benthic fish species *Notothenia coriiceps*, which feeds primarily on krill^20^, underscore the importance of krill for these regions. Of the total krill stock in the Southern Ocean, the majority live over deep oceanic water^42^, suggesting that the increasing discharge of glacial meltwater and particles into the coastal zones of the WAP might not affect the entire krill stock. However, summer (when glacier melting and sediment input are highest) is a critical time of the year for krill, particularly in terms of the energetic demand to fuel growth and reproduction^43^. In summer, cross-shelf gradients result in a higher abundance of krill inshore and over the continental shelf^44^,^45^. For example, some inshore regions, such as the Bransfield Strait, experience pulses of increased faecal flux associated with high populations of krill^46^. Thus, due to the central role of krill in these regions, an increasing loss of krill would have a large impact on the functional biodiversity of coastal systems. If krill in coastal areas are increasingly affected by sediments and mortality removes a proportion of them from the water column, birds and mammals relying on krill will have higher energetic costs associated with fulfilling their nutritional demands; this effect has been observed for species such as the Adélie penguin^47^, the abundance of which has changed in the area around Potter Cove. In addition, the strong decrease in krill biomass since the mid-1970s has been attributed to the decrease in winter sea ice cover^33^. Other investigations have demonstrated that krill appear to be sensitive to increasing seawater temperature^48^ and ocean acidification^49^. However, the krill’s performance window with regard to stressors related to anthropogenic warming is far from clear. The increasing threat to krill due to increasing glacier melt might be not be significant in the context of the current total stock, but in the long term, it is one additional threat among a number of stressors caused by climate change, which will increase and, in combination with other factors, will most likely impact the stock in the future.

## Materials and Methods

### Krill mortality events

Mortality events of krill were observed on several occasions on the southern shore of Potter Cove (Table 1). The first event was detected in 2003, and from 2003 to 2012, monitoring of the coastline in the vicinity of Carlini Station was performed when the cove was free of ice. It should be noted that more events may have gone undetected during the years of the study. Quantification of the number of dead animals was not always possible; therefore, a presence-absence matrix was constructed using qualitative categories for abundance as follows: 0 (absence of krill), 1 (abundances from 10 to 10^2^ krill m^−2^) and 2 (>10^2^ krill m^−2^). For the strandings, all organisms along a 10 m × 1 m transect (2003) and within a 1 m × 1 m square (2007) were counted and classified as adults, juveniles or larvae. In some of the events, other planktonic organisms were identified but not quantified; those data are not presented here. An absence of krill was recorded for dates during the period in which stranding events were studied when the water column was sampled and no stranded krill were found.

### Environmental data

Fifteen krill stranding events were recorded. Environmental information for seventeen samplings during the study period in which no stranding event occurred is included in the analyses. Meteorological data were supplied by the Servicio Meteorológico Nacional (SMN) of the Argentinean Air Force at Carlini Station, and tidal heights were provided by the Servicio de Hidrografía Naval (SHN) of Argentina. The mean and maximum wind speed for the 24 h preceding the sampling date and the predominant direction of the strongest wind were determined. The average air temperature was calculated for the week preceding the sampling events. In addition, the positive degree-days (PDDs) was estimated by adding the positive daily average temperatures for the previous month to the sampling event.

The mean values of the oceanographic parameters were used for the integrated water column. Because meteorological and wind conditions during the stranding events usually prevented oceanographic sampling, the closest date for which information was available was used. When the closest date was more than 10 days before the stranding event, no data were considered available. The oceanographic data are part of a long-term monitoring program that started in 1991, in which the water column in Potter Cove is sampled weekly in the summer and biweekly in the winter, whenever the meteorological conditions allow sampling. A SeaBird SBE Conductivity-Temperature-Depth profiler (CTD; SeaBird Electronics) was used to record seawater temperature and conductivity (transformed to salinity). However, water temperature and salinity were not included in the analyses because insufficient data were available for the studied dates. Water samples were collected at five depths (i.e., 0, 5, 10, 20, and 30 m) with 4.7 L Niskin bottles. For Chl-*a* analysis, seawater (0.25–2 L) was filtered through 25 mm Whatman GF/F filters under gentle vacuum and dim light. Photosynthetic pigments were extracted in 90% acetone for 24 h at 4 °C in the dark. Extract absorbance was read using a Shimadzu RF-1501 spectrophotometer, and concentrations were calculated and corrected for phaeopigment content following the method of Strickland and Parsons^50^. The total suspended particulate material (TSPM) concentration was measured gravimetrically after filtering 0.25–2 L of seawater through combusted pre-weighed 25 mm Whatman GF/F filters. After filtration, the filters were rinsed twice with distilled water to remove salts and then dried for 24 h at 60 °C and weighed again. All environmental data are available at http://doi.pangaea.de/10.1594/PANGAEA. The relation between the proposed explanatory variables and the number of stranding events was analysed by means of Generalized Linear Models (GLMs) using a Poisson error distribution family and a log-link to ensure only positive model predictions. To find the optimal model, we followed a backward selection criterion, where the process ended when all the variables retained during the selection were significant. The analysis of the relation between TSPM and the other environmental variables followed the same approach, using a Gaussian error distribution family (generalized linear model). To visualize the effects of environmental variables over the modelled probability of mass strandings (from the GLMs), the *visreg* function (visreg package) was used^51^. Partial effects reflect the effect of a particular environmental factor on the response variable (mass stranding category) holding all other variables constant (in this case, the median value of the numerical variables and the most common category were used as factors).

MODIS-Aqua spatially extracted Level-2 files were acquired for the study area from the NASA ocean color web page (http://oceancolor.gsfc.nasa.gov). Two images (January 16, 2013 and January, 16 2014) were chosen because of their relatively low cloud cover over the area of interest.

The standard Kd, the diffuse attenuation coefficient, product derived from Kd2 algorithm^52^ was obtained. This Kd product was updated using *in situ* data from NOMAD version 2. The algorithm form describes the polynomial best fit relating the log-transformed geophysical variable to a log-transformed ratio of remote-sensing reflectance (Rrs) as Rrs_(490 nm)_/Rrs_(555 nm)_. Kd was mapped to a WGS 84 reference system (datum WGS84, ellipsoid WGS84 at 1100 m of spatial resolution at nadir and co-registered with respect to a reference landmask), and used as a proxy for the presence of inorganic particles in the water column^33^. Land and cloudy pixels were flagged to zero.

### Feeding and absorption efficiency experiments

Juveniles and adults of *E. superba* were collected from the outer part of Potter Cove using a 200 μm mesh Nansen net with a 2 L cod end, towed vertically (100 m below the surface) and obliquely with a winch installed on a Zodiac rubber raft. The cod end was immediately transferred to a 50 L plastic bucket filled with filtered seawater and then immediately transferred to a cold room (0 °C) where the animals were placed in a 100 L container.

A Sartorius MC1 balance (precision = 10^−4 ^g) was used to determine wet weight. To estimate dry weight, animals were placed on aluminium foil and dried at 60 °C for 3 days and then weighed again. The organic content was determined by ashing the tissues at 500 °C for 4.5 h and calculating the difference between wet and dry weights. Faecal pellets were processed in the same manner as krill.

### Feeding experiments

A natural phytoplankton culture was used as food for the experimental incubations. Phytoplankton were collected using 5 L Niskin bottles. The content of the bottles was transferred to 30 L aquaria (located in the cold room at 0 °C) previously filled with filtered sea water. A series of 5 phytoplankton cultures were established, and the Chl-*a* concentration was measured every second day to track phytoplankton growth. A series of three experiments were performed to estimate feeding rates. The first experiment was performed on increasing phytoplankton concentrations from the cultures describe above. The phytoplankton were diluted in filtered seawater to attain the various experimental concentrations, without any added sediment. This dilution process allowed determination of the daily ration (see below) for each phytoplankton concentration. The second experiment used a fixed phytoplankton concentration (based on the most typical field Chl-*a* concentrations for the area^2^), to which different concentrations of particulate matter (0.1, 10, 40, 80 and 100 mg TSPM L^−1^) were added, reflecting the range of *in situ* concentrations recorded in Potter Cove^1^,^10^. The added particles were obtained from natural sediments taken from the seafloor of the inner cove with a Van Veen grab sampler at a 20 m depth, near a creek mouth. Sediments were dried at 70 °C and sieved through a 50 μm mesh sediment sieve. This size fraction was chosen because it could be easily resuspended in seawater. The organic fraction of the sediments was determined by ashing the filtered material for 4 h at 400 °C and then re-weighing the sample. The average proportion of organic matter was 2.36 ± 0.07%. Experiments were run on adult (45.22 ± 0.44 mm length, 515.46 ± 159.52 mg DW, 257.73 ± 79.76 C content) and juvenile (26.57 ± 0.71 mm length, 36.81 ± 2.22 DW, 18.40 ± 1.11 C content) krill. The total length of krill (mm) was measured from the front of the eye to the tip of the telson. Four juveniles were placed in each of 3 replicate 2.4 L bottles. The bottles were installed in a plankton wheel to maintain the food and sediments in suspension and ensure an equal concentration in all the bottles. For adult krill, 10 organisms were placed in 60 L containers equipped with a gentle mixing system to ensure that the food and the sediments always remained suspended. The experiment was run for 24 h. Once completed, animals were lyophilized for 24 h, and individuals were weighed and ground to powder in liquid nitrogen. For analysis of C, 0.2–0.5 mg aliquots of each krill homogenate were analysed^15^,^50^.

A similar experiment was run with juveniles, comparing two different natural waters from Potter Cove, designated as brown and clear waters. Brown water contained 17 mg L^−1^ TSPM and 0.9 μg Chl-*a* L^−1^, while clear water contained 10 mg L^−1^ TSPM and 1.3 μg Chl-*a* L^−1^. Four juveniles were placed in each of 3 replicate 2.4 L bottles. The experiment was run for 24 h.

All experimental conditions were maintained at a temperature of 0 ± 0.48 ° C and salinity 34 ± 0.3.

Chl-*a* was analysed on 3 replicate 250 mL samples from each container at the beginning and after 24 h incubation^29^,^53^. Feeding was estimated as clearance rate (CR, ml mg^−1^ body C h^−1^). No significant changes in Chl-*a* concentration were detected in the controls (no krill), so that CR was calculated as^54^:


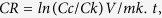


where *C*c and *C*k are the initial and final Chl-*a* concentrations, respectively, *V* is the volume of the container (ml), *mk* is the body mass (mg C) of the krill, and *t* is the duration of the experiment (h). Ingestion rates (IR) were calculated as the product of CR and the initial carbon concentration (mg ml^−1^) as ***IR*** = ***CR.Ci*** and then expressed as the daily carbon ration (% body C d^−1^) under the assumption that krill feeding rates reflect the daily average rate.

The depletion of autotrophic biomass ranged between 1 and 20% in all experiments.

Single factor, one way ANOVA was used to evaluate the effects of TSPM on the daily carbon ration for both, adult and juvenile individuals of *E. superba*. When significant (*p* < 0.050) differences were detected post-hoc Tukey HSD tests were run. The daily carbon ration was log (+0.5) transformed to pass homogeneity and normality assumptions^55^. Analyses were run under the free statistical software R, version 2.15.1^56^.

### Absorption efficiency experiments

The effect of the concentration of particles on the absorption of organic matter and the production of faeces was investigated using natural seston as a food and two different concentrations of fine sediments. The organic fraction in the added sediments was 1.87 ± 0.1%. Three experimental conditions were used: 1) no added sediment (TSPM = 2.39 ± 0.5 mg L^−1^), 2) natural seston + 20 mg L^−1^ sediment (TSPM = 22.3 ± 1.6 mg L^−1^), 3) natural seston + 40 mg L^−1^ sediment (TSPM = 42.8 ± 2.3 mg L^−1^). Prior to the experiments, animals were starved during 48 h in GF/F filtered seawater to empty their stomachs. Six cylindrical 8 L aquaria with individual recirculation pumps were settled in a running 90 L sea water flow bath to keep water at *in situ* temperatures (0 ± 1 °C). A 200 μm mesh was fitted 10 cm above the bottom of each aquarium to allow water to flow and avoid the disruption of the faecal pellets in the circulating system. One control (no krill) and five replicates with 12 animals each (size range: 35–46 mm total length) were used for each experiment. Incubations lasted for 24 h, and no dead animals were registered in any of the experiments. There was no significant difference between initial and final TSPM concentrations in the control aquaria among different experimental trials, and therefore a correction for sedimentation of particles was not needed.

The absorption efficiency was estimated using the Conover ratio^57^, which assumes that only the organic component of the food is significantly affected by the digestive process. Thus, the absorption efficiency was obtained as the difference of the ratio of mass loss after food combustion, and the corresponding percentage of mass loss after combustion of faeces: Absorption efficiency 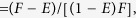 where *F* is the organic fraction in the food and *E* is the organic fraction in the faeces. The absorption efficiency was then reported as percentage. The time elapsed between food delivery and faeces production was recorded. The biodeposition rate was calculated as the dry weight of faeces produced per individual per day. The weight-specific biodeposition rate was referred to animal body mass per day. The faeces were collected every 30 minutes during the first 6 h of incubation and then every hour; the faeces were pooled for absorption efficiency calculations.

Differences among treatments were analysed using one-way ANOVA, since all data met the assumptions of ANOVA analysis. Normality of residuals was tested by mean of Shapiro-Wilks test^58^, while homoscedasticity was confirmed by Levene’s test^59^. When significant differences were encountered, the Tukey–Kramer method^60^ was used as a post-hoc test to identify significant differences among the means. Statistical analyses were run with the softwares InfoStat v.^61^ and PAST v. 3.04^62^.

### Krill stomach content analyses

Freshly dead or dying krill (referred in the text as ‘dead krill’) were collected along the coastline and from shallow coastal waters during the mortality events of 2008 and 2009. Samples were blotted dry and stored at −80 °C for stomach content analyses at the Alfred Wegener Institute, Helmholtz Centre for Polar and Marine Research (Bremerhaven, Germany). During those years, zooplankton and water samples were collected from a rubber boat at the inner part of the cove. Healthy krill (referred in the text as ‘living krill’) was caught only once during this period. The stomach content was compared with that from dead krill. Ten adult krill individuals sampled during each of the stranding events and of the catchment were studied (n = 40). Wet body weight was registered before dissection. Then, the exoskeleton was removed and the stomach was taken out and weighed. Stomach contents were analysed^29^ and an Utermöhl chamber was used to count and identify the particles. Abundant size classes (>100 particles) were counted in two perpendicular transects across the chamber, while for the less abundant size classes the whole chamber was counted at 250×. The dimensions of the different items in the stomachs were measured and their biovolume calculated^63^,^64^. The lithogenic particles found in the stomach contents were enumerated and grouped into size intervals according to their length as follows: <3.8, 3.8–7.5, 7.6–18.8, 18.9–37.5, 37.6–56.3, 56.4–75.0 and >75.0 μm. The length of each particle was measured and its volume estimated assuming a rectangular parallelepiped form with width and height half their length.

The wet weights and the composition of the stomach content did not follow a normal distribution, and therefore medians and percentages were calculated to compare the data among the three events in which stomach content was studied by means of Kruskal-Wallis test. When no differences among strandings were found, data were grouped and compared with the results from living krill by means of Mann-Whitney’s test.

For the scanning electron microscope (SEM) analyses, the stomach and the hindgut were removed from the frozen krill, and their contents released separately in Eppendorf tubes. The samples were prepared^65^, with certain modifications. Briefly, each of the stomach contents was washed 5 times with deionized water to remove salt. Then, the sample was cooked in NaOH 1N at 60 °C during 2 h to remove the rest of stomach and gut tissues, and washed again 5 times with deionized water. The sample was then filtered on 0.1 μm cellulose nitrate membrane filter and air-dried. Filters were mounted on SEM stubs and sputtered with gold-palladium (ca. 20 nm thickness). Images were taken with a FEI Quanta 200 FEG microscope.

## Additional Information

**How to cite this article**: Fuentes, V. *et al.* Glacial melting: an overlooked threat to Antarctic krill. *Sci. Rep.* **6**, 27234; doi: 10.1038/srep27234 (2016).

## Supplementary Material

Supplementary Information

Supplementary Information

## Figures and Tables

**Figure 1:**
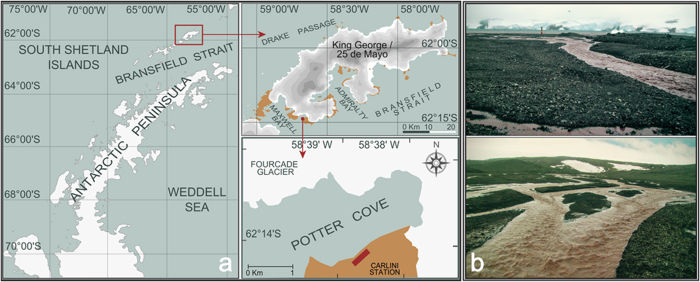
(**a**) Map showing the location of the study area in the vicinities of Potter Cove, King George/25 de Mayo Island, South Shetland Islands, Antarctica during March 2007; maps were created using Inkscape 0.48.5 (URL: https://inkscape.org/). (**b**) Typical glacier-originated melt-water creek carrying large amounts of particles. Credit photos: V. Fuentes.

**Table 1:** Environmental variables corresponding to the periods in which krill strandings were studied.

	Category	Air temp. ( °C)	PDD ( °C)	Wind speed (kn)	Wind direction	Maximum tidal amplitude (m)	Water temp. ( °C)	TPSM (mg L^−1^)	Chl-*a (μg L^−1^)*
5-Feb-03	0	3.0	20.7	4.4	W	1.53	nd	28.55	0
14-Feb-03	0	2.7	18.8	8.0	E	1.64	nd	4.85	0.32
17-Feb-03	0	3.1	21.4	3.2	W	2.06	nd	30.5	0.99
19-Feb-03	0	3.2	22.2	9.2	SW	1.98	nd	26.5	0.33
24-Feb-03	2	3.4	23.5	12.4	NW	0.44	nd	10.65	1.2
5-Mar-03	0	−2.2	0.0	7.6	E	1.6	nd	11.05	0.8
18-Mar-03	0	−2.0	0.0	14.1	SE	1.94	nd	27.82	0.59
24-Mar-03	2	0.8	7.8	5.3	W	0.65	nd	3.36	0.29
4-Apr-03	1	1.5	10.6	26.6	NW	1.23	nd	nd	nd
9-Apr-03	0	0.3	12.7	12.5	E	0.39	nd	3.16	nd
6-Mar-06	1	2.2	15.5	17.1	SE	1.75	nd	nd	nd
16-Oct-06	1	−0.5	5.0	5.5	E	0.62	nd	nd	0.17
1-Dic-06	1	1.3	9.3	5.4	SE	0.56	nd	nd	nd
14-Jan-07	0	2.9	20.4	7.9	W	0.08	nd	1.98	0.76
22-Jan-07	0	2.2	15.2	3.6	SW	1.38	nd	4.27	1.09
25-Jan-07	1	2.9	20.0	5.0	W	1.46	nd	10.25	1.09
1-Feb-07	0	2.8	19.8	20.1	E	1.76	nd	4.39	0.46
16-Feb-07	1	3.1	21.5	6.4	SW	1.92	nd	10.50	1.07
21-Feb-07	1	2.1	14.9	7.6	SE	1.47	nd	15	1.07
1-Mar-07	0	0.7	7.5	6.7	NW	1.43	nd	2.76	0.23
15-Mar-07	0	−2.9	1.3	11.3	NW	0	nd	2.56	0.49
25-Mar-07	2	−0.4	2.5	10.5	W	0.69	nd	7.5	nd
30-Sep-08	2	1.6	11.3	37.3	NW	1.87	nd	nd	nd
14-Oct-08	0	−0.3	2.8	4.5	W	1.81	−0.96	2.8	0.56
27-Oct-08	2	0.9	6.2	16.0	N	1.63	nd	3.6	0.85
30-Oct-08	0	1.2	8.7	19.8	NW	1.95	−0.25	2.64	nd
16-Mar-09	0	2.9	20.1	20.1	W	0.9	1.61	8.2	0.99
28-Mar-09	2	2.1	14.7	21.7	NE	1.36	nd	16.8	nd
27-Jan-12	0	2.1	14.6	9.4	W	1.53	1.61	0.007	1.88
16-Feb-12	1	2.5	17.3	13.3	W	0	nd	nd	nd
18-Feb-12	1	1.4	12.0	26.9	SE	0.37	nd	nd	nd
2-Mar-12	0	2.5	17.3	17.0	NW	0	1.7	0.016	18.06

Category 0 indicates the dates when no dead krill were observed on the beach. Category 1 indicates dead krill present at <100 ind m^−2^. Category 2 indicates mass strandings of >100 ind m^−2^. See text for methodological considerations of the variables shown. PDDs: positive degree-days; TSPM: total suspended particulate matter; Chl *a*: chlorophyll *a*; nd: no data available.

**Table 2:** Characteristics of krill from the 2008–2009 mortality events and of living krill: median (range) body and stomach wet weights (mg) and volume of total diatoms (μm^3^), total lithogenic particles, and lithogenic particles >1.1 10^5^ μm^3^.

	DEAD KRILL			LIVING KRILL
	30 Sept 2008	27 Oct 2008	28 Mar 2009	21 Dec 2009
Body wet weight (mg)	592.5 (390.6–964.2)	765.8 (493.1–1026.1)	784.3 (686.2–1016.9)	742.3 (491.8–1054.2)
Stomach wet weight (standardized, mg)	0.007 (0.004–0.011)	0.008 (0.006–0.009)	0.006 (0.004–0.009)	0.011 (0.005–0.011)
Total volume of diatoms (μm^3^)	0.023 (0–12.242)	0.001 (0–0.004)	0.008 (0–0.077)	0.274 (0.084–54.028)
Total volume of lithogenic particles (μm^3^)	0.113 (0.014–0.194)	0.085 (0.018–0.170)	0.029 (0.009–0.146)	0.049 (0.028–0.075)
