# Supplementary material for: Glacial melting: an overlooked threat to Antarctic krill
Source: Sci Rep. 2016 Jun 2;6:27234. doi: 10.1038/srep27234 (PMC4890292; doi:10.1038/srep27234)
Supplement: Supplementary Information [file srep27234-s2.pdf]

Glacial melting: an overlooked threat to Antarctic krill

Verónica Fuentes, Gastón Alurralde, Bettina Meyer, Gastón E. Aguirre, Antonio Canepa, Anne-Cathrin Wölfl, H. Christian Hass, Gabriela N. Williams, Irene R. Schloss,

Supplementary material: Video taken on March 2007 of krill feeding in a particle-laden water column. Note the colour of the digestive tract of the animals. Credits: V. Fuentes and A.-C. Wölfl.
